# Comparison Between the Bone Lid Technique and the Traditional Technique in Surgical Treatment of the Posterior Mandibular Lesions: A Randomized Controlled Trial

**DOI:** 10.7759/cureus.26223

**Published:** 2022-06-22

**Authors:** Mohamad Husam Abu hawa, Zaed Shehri, Issam Alkhouri

**Affiliations:** 1 Oral and Maxillofacial Surgery Department, University of Damascus Dental School, Damascus, SYR

**Keywords:** bone defect healing, cyst enucleation, posterior mandible, buccal cortical plate, piezoelectric, bone window, bone lid technique

## Abstract

Introduction

Cystic lesions of the jaws and the impacted teeth are two of the most common cases that require surgical intervention in oral and maxillofacial surgery; however, surgeons also frequently use a traditional technique that involves the removal of the buccal bone plate. This study was conducted to compare the clinical and radiologic outcomes of the bone lid technique and the traditional technique.

Methods

This randomized controlled trial included 20 patients who were randomly divided into two groups (n = 10 each): the T group, in which the lesions were accessed using the traditional technique with classical rotating instruments, and the BL group, in which the lesions were accessed with the bone lid technique performed using a piezoelectric device, with repositioning of the buccal bone plate. Operative time, pain, edema, inferior alveolar nerve injury, and bone defect healing were measured during clinical and radiological follow-ups at 24 h, 72 h, one week, one month, and six months after the surgery.

Results

Normal soft tissue and bone healing were observed in all cases except one case in the BL group. The T group had a shorter mean operative time than the BL group. In terms of pain, edema, and inferior alveolar nerve injury, the groups did not differ statistically significantly. The percentage of bone defect healing was significantly greater in the BL group than in the T group after six months of follow-up.

Conclusion

The bone lid technique performed using a piezoelectric device was effective and safe for managing lesions in the posterior mandibular region and was not associated with increased postoperative complications. The disadvantages of this technique include a longer operative time and the need for fixation tools in some cases. In contrast, this technique outperforms the traditional technique in terms of reducing bone loss and improving the healing of bone defects.

## Introduction

Cystic lesions of the jaws and the impacted teeth are two of the most common cases that require surgical intervention by oral and maxillofacial surgeons [1]. Jaw cysts can be treated with either surgical or nonsurgical treatment. For inflammatory periapical lesions with an insignificant size and extent, conservative nonsurgical methods should be applied [2]. Cystic lesions can be surgically treated by cystectomy, cystotomy, or a combination of both. A surgical extraction is the most commonly used treatment for impacted teeth [3].

For lower jaw surgical treatment, surgeons frequently use a traditional technique that involves the removal of the buccal bone plate to allow for visual and surgical access to the lesion; however, this approach has several complications, particularly in patients with large amounts of bone loss. Moreover, the mandibular canal and the presence of vital teeth may make surgical access to the lesion challenging [4].

The main disadvantage of this method is enucleation-induced bone loss, which may cause difficulties with securing dental implants in this area if needed in the future. Although numerous types of bone and connective grafts provide satisfactory solutions to these problems, some patients may not want to undergo a second surgical intervention or bear the expenses associated with these procedures [5]. Surgical interventions for alveolar bone disease result in bone defects owing to both the disease itself and the need to perform osteotomies to make the lesion visually and surgically accessible [6].

Endoscopy to enucleate jaw cysts [7] and the so-called “bone lid technique” have been proposed as alternatives to ostectomy [8]. The bone lid technique is aimed at avoiding the formation of significant bone defects, providing better intraoperative visualization, providing better support for the mucoperiosteal flap, and promoting bone regeneration after healing [4,9] while taking into consideration that the features of the bone defect may affect bone healing [10].

The bone lid technique was originally proposed for apicoectomies; it required cutting a window into the bone, creating a lid, removing it, accessing the relevant location, replacing the lid to its original position at the end of the surgery, and fixing or not fixing the lid, depending on the necessity [5].

Long shank drills [11], microsaws [5], or piezoelectric devices can be used to perform bone lid osteotomies. Piezoelectric surgery has been reportedly used to design bone lids to ensure precise, thin osteotomy margins, thus reducing bone loss and facilitating lid relocation [9,12].

This trial aimed to compare the clinical and radiologic outcomes of the bone lid technique performed using a piezoelectric device versus the traditional technique in patients requiring extraction of the bony lesions in their posterior mandibular region.

## Materials and methods

Study design

This randomized controlled trial was performed at the Oral and Maxillofacial Surgery Department, University of Damascus Dental School, Syria, between June 2019 and August 2021. The study was approved by the Scientific Research Committee at the University of Damascus Dental School (UDDS-708-28082018) and the Local Research Ethics Committee (309-24092018/SRC-3960). The study was funded by the University of Damascus Dental School Postgraduate Research Budget (Reference number: 80015489287DEN) and all the clinical case was made according to the Helsinki Declaration of Ethical Principles.

Patients

**Figure 1:**
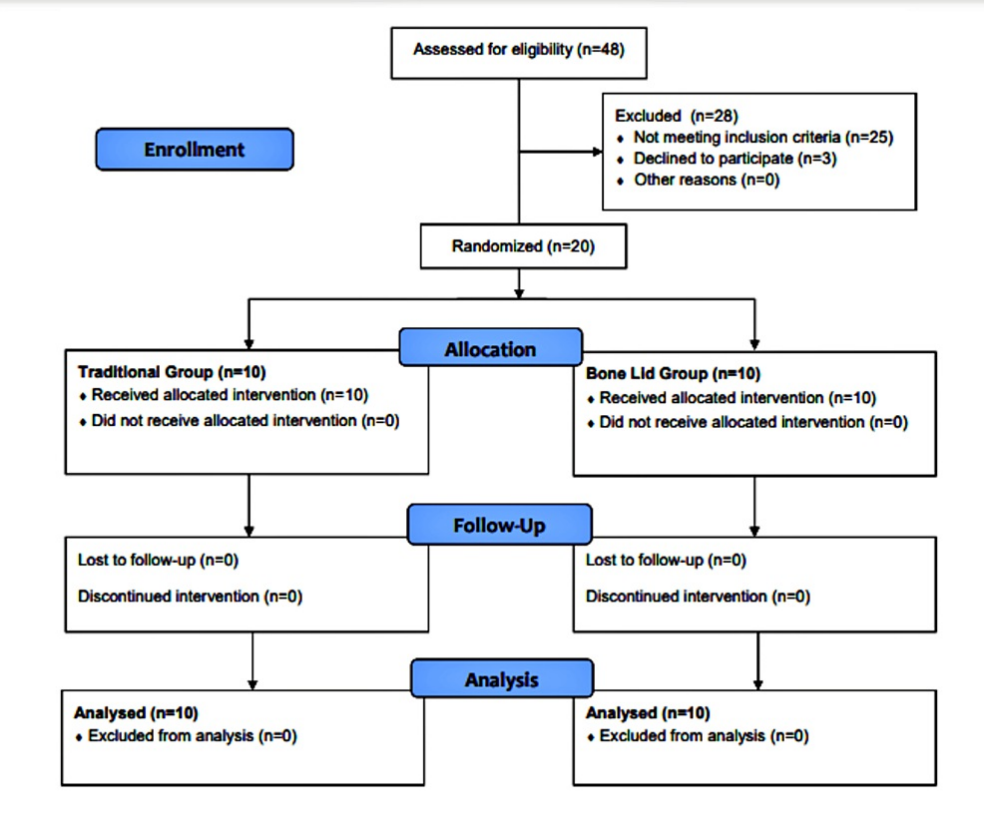
CONSORT flow diagram of patients' recruitment, follow-up, and entry into data analysis. CONSORT: Consolidated Standards Of Reporting Trials

In total, 20 patients (11 women and 9 men) aged 18-45 years (mean age, 33.4 years) were included. The inclusion criteria were as follows: age >18 years, need for the removal of a bony lesion (cysts, benign tumors, or impacted teeth) located in the posterior mandibular region, lesion size (diameter) ≥1 cm, and the existence of a normal residual buccal cortical plate with the adequate thickness (≥1 mm). Moreover, all included patients provided written informed consent and completed clinical and radiological follow-up visits for six months after the surgery. A CONSORT (Consolidated Standards Of Reporting Trials) flow diagram of patients' recruitment, follow-up, and entry into data analysis is shown in Figure 1.

The exclusion criteria were as follows: patients with blood coagulation disorders or systemic diseases that could interfere with healing, patients taking medications that affect bone metabolism, those who underwent head and neck radiotherapy in the last 12 months, pregnant patients, and patients who were heavy smokers (≥40 pack-years).

The included patients were randomly divided into two groups; each group comprised 10 patients. For the traditional group or T group, the lesions were accessed using the traditional technique with classical rotating instruments, whereas for the bone lid or BL group, the lesions were accessed using the bone lid technique with a piezoelectric device.

Randomization, allocation, and blinding

Patients were assigned to the bone lid group (BL) or the traditional group (T) with an allocation ratio of 1:1 using a simple randomization technique. Each patient was asked to select a folded piece of paper from a box containing 20 pieces of paper; on 10 pieces, the word "BL group" was written, while the word "T group" was written on the other 10 pieces. The patient was assigned to one of the two groups according to the selected paper. A member of the academic staff not involved in the study project was asked to perform the random allocation sequence generation and participants' enrollment. Blinding of the patients and practitioners was not applicable. Therefore, blinding was applied only for data analysis.

Preparatory procedures

The patients’ preoperative data, demographic information, and medical history were obtained. Complete clinical, extraoral, and intraoral examinations were then conducted. Each patient underwent cone-beam computed tomography (CBCT) scanning, which was performed using a 3-dimensional imaging system (PaX-i3D Green, Vatech Co., Hwasung, Korea).

Surgical procedures

**Figure 2:**
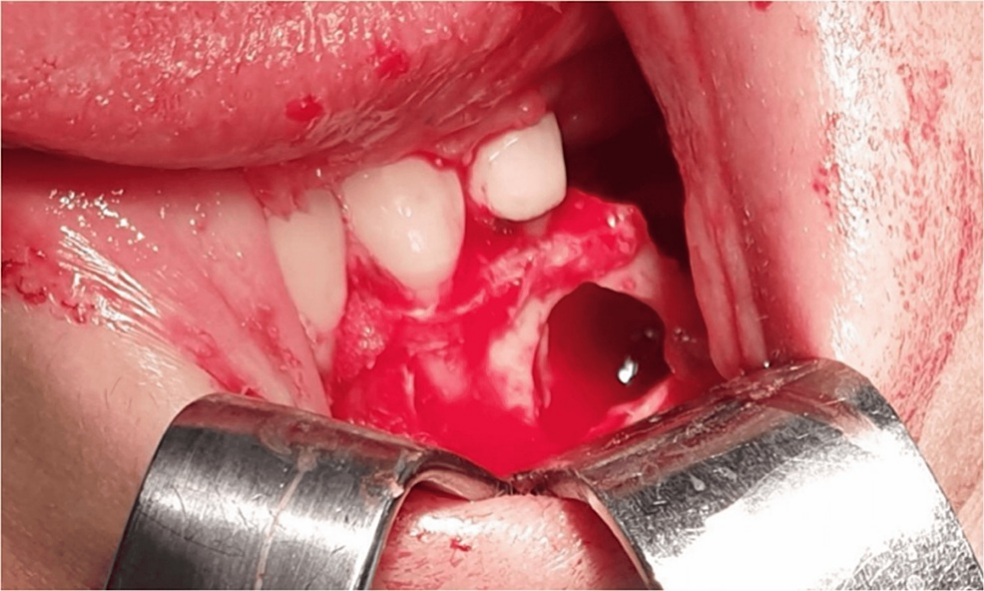
The bone defect after intervention using the traditional approach.

All procedures were performed with the patients under conscious sedation and local anesthesia. A full-thickness flap of adequate size and design was elevated such that it reached the buccal bone over the lesion, depending on the area. In the T group, the lesion was accessed after the removal of the surrounding buccal bone using classical rotating instruments (Figure 2).

**Figure 3:**
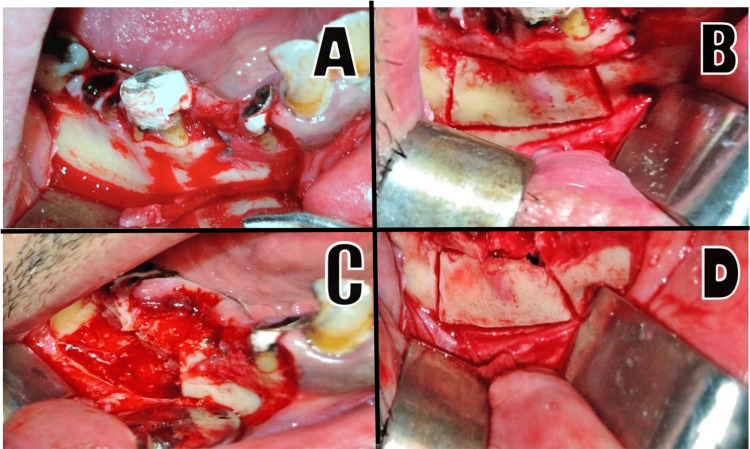
Images of a patient from the BL group. A: Elevating the full-thickness flap. B: Using piezosurgery to create a bone lid. C: Removal of the bone lid, providing entry to the lesion; teeth 46–47 were extracted during the surgical process, and the inner lesion was enucleated. D: The bone lid was restored to its original position, without the need for fixation tools.

In the BL group, a bone window was created using a piezoelectric device with osteotomy tips (piezosurgical inserts: OT7, OT8L, and OT8R; Mectron Medical Technology, Carasco, Italy). The bony window created was at least 4-6 mm larger than the original, radiographically pre-evaluated size of the lesion. The piezosurgery tip was directed through the normal buccal cortical plate and down to the trabecular bone at an angle. Using an angled chisel, the bony lid was gently freed and placed in a physiological saline solution. Next, the inner lesion was completely enucleated, the bone lid was restored to its original position (Figure 3), and the stability of the lid was checked. If necessary, the bone lid was fused with the adjacent bone using a 2/0 resorbable suture (polyglycolic acid; Shandong Haidike Medical Products Co., China).

In both groups, at the end of the procedure, the mucoperiosteal flap was replaced and sutured using 3/0 black silk sutures (Kaihong Healthcare Co. Ltd., Nanjing, China), and the operative time was recorded. If required, surgical specimens were sent to a pathologist for histological examination. Postoperative medication included amoxicillin + clavulanic acid (1 g/125 mg) twice a day for seven days, ibuprofen (400 mg) thrice a day for five days, and chlorhexidine mouthwash (0.12%) thrice a day for two weeks.

Outcome measures and clinical assessment

All patients underwent surgical evaluation 24 h, 72 h, one week, one month, and six months after the surgery. Postsurgical pain was measured using the visual analog scale (VAS) [13]. Postsurgical edema was assessed using the three-line method, which involves measuring three lines between five different points: the tragus, external corner of the eye, soft tissue pogonion, angle of the lower jaw, and lateral corner of the mouth. The difference between the preoperative and postoperative measurements was indicative of the facial edema on each day [14]. Periodontal probing was performed on the lower lip (nociception) to assess possible sensory disturbances relative to the opposite side [15]. Soft tissue healing was evaluated at each follow-up visit, and signs of inflammation, suppuration, necrosis, or bone exposure were documented.

Radiographic assessment

Notably, CBCT was performed six months postoperatively, and the findings were compared with preoperative CBCT findings to assess the bone defect and bone lid healing. Radiographic assessment was performed by three experienced observers (two oral and maxillofacial surgeons and one oral medicine specialist) using a software for the analysis and processing of radiographic images (Ez3D Plus 2009; Vatech Co., Hwasung, Korea)

**Figure 4:**
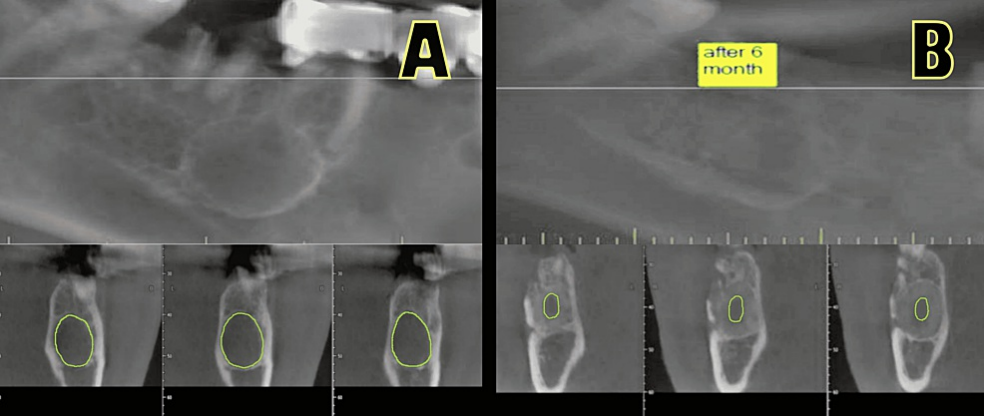
Radiographic assessment. A: A well-defined radiolucent lesion present apically to tooth 46 is visible on the presurgical one-beam computed tomography (CBCT) scan. lesion covered in the buccal cortical plate. B: CBCT scan, six months after the surgery. Good bone defect filling was noted, and the bone lid is integrated.

Residual radiolucent areas and postextraction defects were measured manually on each coronal section of the postoperative images using the area tool embedded in the software. Preoperative and postoperative findings were compared to determine the healing of the bone defect and express it in percentages (Figure 4).

Statistical analysis

SPSS® software (version 24.0; IBM Corp., Armonk, NY) was used for recording and statistically analyzing the data. The Kolmogorov-Smirnov test was used to check if the data had a normal distribution. In terms of operative time, pain, edema, and the percentage of bone defect healing, an independent t-test was used to compare the results between both groups. The Chi-square test was used to compare the incidence of inferior alveolar nerve (IAN) injury between the two groups. A P-value of <0.05 was considered statistically significant at the 5% level.

## Results

The present study involved 20 patients with bony lesions or impacted teeth in the posterior region of the mandible who were indicated for surgical treatment. The selected patients were randomly divided into two groups as follows. The T group comprised 10 patients (six with a cyst, two with impacted teeth, one with an endodontic lesion, and one with an odontoma). The mean diameter of the lesions was 15.6 mm. The BL group comprised 10 patients (six with a cyst, two with impacted teeth, and two with endodontic lesions). The mean diameter of the lesions was 13.9 mm.

Clinical outcomes

All soft tissues were identified as healed by primary intention at one month postoperative clinical follow-up. We observed no signs of inflammation, necrosis, or suppuration, except in one patient in the BL group. This patient showed evidence of bone exposure and bone lid integration failure, because of which the lid was removed during revision surgery (three weeks postoperatively). In the BL group, fixation tools were not used in eight out of 10 cases, and resorbable sutures were required in two cases. Table 1 shows the clinical results obtained.

**Table 1:** Clinical results

Patient number	Treatment method	Lesion type	Operative time (min)	Pain (visual analog scale scores)			edema		Inferior alveolar nerve injury
				After 24 hours	After 72 hours	After 7 days	After 24 hours	After 7 days	
1	Traditional	Residual cyst	55	7	7	4	2.3	0.5	No
2	Traditional	Traumatic cyst	62	7	6	3	2.4	0.2	No
3	Bone lid	Radicular cyst	85	8	5	3	3.1	0.6	No
4	Bone lid	Residual cyst	92	8	5	3	2.9	0.6	No
5	Bone lid	Residual cyst	102	7	4	2	2.4	0.4	No
6	Traditional	Radicular cyst	46	6	4	2	2.6	0.4	No
7	Traditional	Peri-apical granuloma	42	8	6	2	2.7	0.3	No
8	Bone lid	Peri-apical granuloma	88	6	3	2	2	0.6	No
9	Traditional	Radicular cyst	49	6	5	2	2	0.2	No
10	Traditional	Impacted tooth	40	6	4	2	2.4	0.3	No
11	Bone lid	Impacted tooth	78	9	6	4	2.2	0.3	No
12	Bone lid	Radicular cyst	94	7	5	2	2.6	0.4	Yes
13	Bone lid	Radicular cyst	86	8	5	2	2.7	0.4	No
14	Traditional	Impacted tooth	42	8	5	3	2.3	0.3	No
15	Traditional	Radicular cyst	56	7	4	3	2.3	0.5	No
16	Bone lid	Radicular cyst	98	8	6	3	2.6	0.3	No
17	Bone lid	Peri-apical granuloma	82	7	5	2	2.3	0.2	No
18	Traditional	Radicular cyst	55	7	3	1	2.9	0.4	No
19	Bone lid	Impacted tooth	95	7	3	1	3.2	0.9	Yes
20	Traditional	odontoma	52	6	4	1	2.6	0.5	Yes

Operative time

The mean operative time was significantly shorter in the T group (49.9 min) than in the BL group (90 min; P < 0.001).

Pain scores

In the T group, the mean VAS score was the highest at 6.8 ± 0.78 (range, 6-8) on postoperative day one. After 72 h, it decreased to 4.8 ± 1.22, and after one week, it further decreased to 2.3 ± 0.94. In the BL group, the mean VAS score was the highest at 7.5 ± 0.85 (range, 6-9) on postoperative day one. After 72 h, it decreased to 4.7 ± 1.05, and after one week, it further decreased to 2.4 ± 0.84. The VAS scores did not significantly differ between groups at any of the three time-points (Table 2).

**Table 2:** Comparison of the visual analog scale scores between the two groups

Follow-up period	Group	Mean	Standard deviation	T-test	P-value
After 24 hours	T	6.8	0.78	1.909	0.072
	BL	7.5	0.85		
After 72 hours	T	4.8	1.22	0.195	0.848
	BL	4.7	1.05		
After 7 days	T	2.3	0.94	0.249	0.806
	BL	2.4	`0.84		

Edema

The three-line method was used to assess postoperative edema in both groups after 24 h and one week. In the T group, the swelling was the highest after 24 h (mean, 2.45) and gradually reduced after one week (mean, 0.36). In the BL group as well, the swelling was the highest after 24 h (mean, 2.6) and gradually reduced after one week (mean, 0.47). No statistical significance was noted in the edema during any of the follow-up periods (Table 3).

**Table 3:** Comparison of edema between the two groups

Follow-up period	Group	Mean	Standard deviation	T-test	P-value
After 24 hours	T	2.45	0.255	1.020	0.321
	BL	2.6	0.389		
After 7 days	T	0.36	0.117	1.468	0.159
	BL	0.47	`0.206		

IAN injury

One out of 10 patients in the T group reported hypoesthesia of the IAN, which persisted for 2 weeks. In the BL group, two out of 10 patients recovered spontaneously (after seven and 28 days, respectively). The Chi-square test was applied to determine between-group differences in terms of IAN injury, and no significant differences were observed between the two groups.

Late radiological outcomes

A CBCT scan was conducted as a part of the radiological follow-up to evaluate bone lid integration and bone defect recovery after six months. The residual alveolar bone defects were assessed to calculate the percentage of bone defect filling; different results were obtained for both groups. The percentage of bone defect filling in the T group ranged from 48% to 78% (mean, 62.7%). This percentage was lower than that observed in the BL group, which ranged from 78% to 96% (mean, 87.1%; Table 4). When the results were compared, a statistically significant difference was noted between the two groups (P < 0.001) in favor of the BL group. In one BL group patient in whom bone lid integration failure was noted, the lesion recovered after revision surgery, which involved the imperfect filling of the bone defect. In the remaining 19 patients, radiological examinations revealed bone healing with no evidence of bone resorption, infection, or lesion relapse.

**Table 4:** Comparison of the percentage of bone defect filling between the two groups

Group	Mean	Standard deviation	Min	Max	T-test	P-value
T	62.7%	9.250	48%	78%	7.12	<0.001
BL	87.1%	5.646	78%	96%		

## Discussion

This study was conducted to compare the clinical and radiographic outcomes of the bone lid technique and a commonly used traditional technique. We restricted the present study to the posterior mandibular region because structural and anatomical differences among different regions of the jaws could affect the outcomes. In addition, the structure of the posterior mandibular region, which comprises a trabecular bone sandwiched between two thicker cortical bony plates [16], makes it easier to remove the bone lid without fracturing it.

In the BL group, a piezoelectric device was used for bone cutting because it gives the surgeon greater control and allows for more precise and fine osteotomies, thereby reducing the amount of missing bone and facilitating the restoration of the bone lid to its original position [12].

The results of this study revealed that the mean operative time in the traditional technique was significantly shorter. This could be attributed to the fact that the bone lid technique involves a greater number of steps and the usage of a piezoelectric device in an osteotomy, which makes it relatively more time-consuming [17].

A well-known clinical benefit of using piezoelectric devices in oral and maxillofacial surgery is that these devices reduce postoperative pain and edema [18-20]. Pappalardo and Guarnieri reported that using a piezoelectric device for the enucleation of mandibular cysts reduces postoperative pain and edema [19]. However, our findings were not in agreement with this report; this disparity could be attributed to differences in the surgical techniques used and because our technique required a longer operation time, knowing that a longer operation time results in greater tissue trauma, and induced a greater inflammatory response [14].

In addition, the bone lid technique also involves the use of chisels, which have been previously reported to increase postoperative pain and edema [21]. Due to these factors, we did not detect any statistically significant differences between the two study groups in terms of postoperative pain and edema, despite using a piezoelectric device in the BL group.

Many researchers have recommended the bone lid technique for the removal of lesions located close to the IAN as it allows for clear visual access, thereby reducing the risk of IAN damage [12,22]. However, in our study, we did not notice a significant difference between the two techniques in terms of IAN injury despite using a piezoelectric device, which has a selective cutting feature [23,24] in the BL group. This could be attributed to the location of the lesions in our study which were at varying distances from the IAN, and the fact that IAN paresthesia could occur because of multiple reasons such as chemical irritation caused by inflammatory mediators or mechanical compression caused by internal bleeding [25,26]. 

After a six-month follow-up period, the bone defect healing was 62.7% in the T group and 87.1% in the BL group, and only one out of 10 patients in the BL group developed bone lid necrosis. The main advantage of the bone lid technique is that it reduces bone loss [5] and preserves the buccal cortical plate, which may be a source of osteoblasts [10]. Repositioning the bone window to its original position reduces the bone defect dimensions and creates a bony barrier that prevents the migration of epithelial tissue into the defect area; therefore, this technique is considered a guided bone regeneration method [5,27,28]. This also clearly substantiates the superiority of the bone lid technique over the traditional technique in terms of bone defect healing, as demonstrated by our study results.

Our results were similar to those reported by Sivolella et al. who assessed bone healing in 11 patients treated using the bone lid technique and reported a bone defect healing rate of 93.8% after 12 months [28]. Moreover, our findings related to bone defect healing differed from those reported in Oh et al.'s retrospective study, perhaps because they only applied the bone lid technique to large cysts and evaluated bone healing using a 2-dimensional panoramic scan [22].

Strengths and limitations of this study

To our knowledge, our study is the first to compare the clinical and radiological outcomes of the bone lid technique and a traditional technique through a randomized controlled trial. However, the short follow-up period, relatively small sample size, and diversity in the type of lesions limit the findings of our current study.

## Conclusions

 The bone lid technique performed using a piezoelectric device was effective and safe for managing lesions in the posterior mandibular region and was not associated with increased postoperative complications. The disadvantages of this technique include a longer operative time and the need for fixation tools in some cases. In contrast, this technique outperforms the traditional technique in patients that have an unaffected buccal cortical plate, patients who are expected to have significant iatrogenic bone loss, and patients who are scheduled for dental implantation after the surgery, because this technique reduces bone loss and improves the healing of bone defects significantly.
